# Foot Arch Changes after Endoscopic Plantar Fascia Release for Recalcitrant Plantar Fasciitis

**DOI:** 10.5704/MOJ.2207.010

**Published:** 2022-07

**Authors:** SK Liew, A Saw, YP Chua

**Affiliations:** 1 Department of Orthopaedic Surgery, Universiti Putra Malaysia, Serdang, Malaysia; 2 NOCERAL, Department of Orthopaedic Surgery, Universiti Malaya, Kuala Lumpur, Malaysia; 3 Department of Orthopaedic, Sunway Medical Centre, Bandar Sunway, Malaysia

**Keywords:** endoscopic plantar fascia release, foot arch, footprint, plantar fasciitis, radiological measurements

## Abstract

**Introduction:**

Endoscopic plantar fascia release (EPFR) is a minimally invasive surgical intervention for recalcitrant plantar fasciitis. Its efficacy has been convincing but the *in vivo* effect on medial longitudinal foot arch and footprint has not been studied. Our objective is to evaluate the changes of foot posture using radiographs and footprints following endoscopic plantar fascia release in recalcitrant plantar fasciitis.

**Materials and methods:**

This prospective cohort involved patients with recalcitrant plantar fasciitis who failed six months of conservative treatment. Two-portal endoscopic release of not more than 50% of plantar fascia width was performed. Footprint and standard weight-bearing anteroposterior and lateral radiographs of the foot were taken pre-operatively and at 12 months post-surgery. Arch index, normalised navicular height truncated, calcaneal inclination angle, calcano-1st metatarsal angle, talonavicular coverage angle and talus-2nd metatarsal angle were measured.

**Results:**

Sixteen patients (18 feet) were reported. Patients’ follow-up ranged from 14 to 31 months after surgery (mean±SD: 23.44±5.76). The increase of arch index, calcano-1st metatarsal angle and reduction of calcaneal inclination angle were found statistically significant (p<0.05). Two normal arch patients progressed to asymptomatic flat arch feet. Three complications were noted between three to nine months post-surgery, one with medial column and two with lateral column symptoms.

**Conclusion:**

There is evidence of reduction in medial longitudinal arch of the foot after EPFR. Although the reduction remains asymptomatic, post-operative complications related to changes in biomechanics of the foot can occur between three to nine months. Patients should be monitored at least for 12 months and longer for those who are symptomatic.

## Introduction

Plantar fasciitis is one of the most common outpatient problems seen in Orthopaedic clinics. About 80-90% of patients responded well with conservative treatment^1-2^. Unfortunately, the time until resolution is often 6 to 18 months, which can lead to frustration for patients^3^. Recalcitrant cases are those with chronic symptoms more than six months despite conservative non-operational management^4^. Endoscopic plantar fascia release (EPFR) has been introduced as an alternative option to conventional open plantar fascia release for managing recalcitrant cases. Alteration of the normal foot biomechanics following plantar fasciotomy or surgical release could have contributed to the decrease in medial longitudinal height, lateral column pain and increasing stress to the second metatarsal bone^5-9^. The minimal invasive surgical technique of EPFR requires less soft tissue dissection and less tissue adhesion, more accurate release of medial plantar fascia compared to open surgical release. Hence, EPFR is believed to be less destructive to medial longitudinal foot arch. Other potential advantages of EPFR include less post-operative pain, shorter hospital stay, earlier return to regular activity and less surgical complications.

Up to date there is no consensus on how much plantar fascia to release to balance between efficacy and complications. Studies showed that complete sectioning of plantar fascia will lead to loss of medial longitudinal arch, destabilise the foot, increase strain to lateral column and forefoot. Barrett *et al*^10^ advocated not more than one third of medial plantar fascia should be released. Cheung *et al*^11^ in their 3-D finite element model biomechanical study suggested that no more than 40% of the plantar fascia should be released, while Brugh *et al*^12^ recommended that 50% should be the upper limit of the case.

Despite these recommendations in the medical literature, there were no clinical or radiological definition of the normal medial longitudinal arch (MLA) height. Various methods have been described to assess the MLA. Direct methods to assess the MLA includes anthropometric and radiographic analysis; indirect methods are through footprint and photographic documentation. Plain radiographs have been regarded as the gold standard for accessing skeletal alignment of the foot in static weight-bearing position^13,14^. Footprint have been used as indirect measurement of arch height since the 1930s^15^. It is a simple, cost-effective yet clinically valid method of foot posture evaluation^16^. Hence, a more comprehensive overview of foot arch changes can be obtained by analysing both radiographs and footprints after EPFR procedure. There were many cadaveric studies on biomechanics changes in the foot following plantar fascia release, but very few were based on radiographic and footprint parameters in live patients.

The purpose of this study is to investigate changes in medial longitudinal arch following EPFR <50% of plantar fascia release based on radiographic assessment and footprint analysis at 1-year post-surgery in a clinical cohort. Our null hypothesis is there is no difference in the analysis of medial longitudinal arch before and after EPFR.

## Materials and Methods

This is a prospective case series study on 16 adults (11 females and 5 males) with 2 cases where both feet were involved (18 feet), diagnosed with recalcitrant plantar fasciitis. The diagnosis was made based on clinical assessment by a senior foot and ankle consultant. The clinical criteria were: (1) Chronic plantar heel pain at least six months, provoked when taking first few steps in the morning/extended period of rest and increases with weight-bearing during the day. (2) Clinically tender at insertion of plantar fascia on medial calcaneal tubercle. (3) No or unsatisfactory improvement after at least six months of non-surgical intervention, which includes physiotherapy, non-steroidal anti-inflammatory drugs (NSAIDs), night splint, local steroid injection, acupuncture, and platelet-rich plasma (PRP) treatment. All patients had minimum six months of non-operative intervention for plantar fasciitis. Local steroid injection patients were given at least three months prior to surgery to ensure maximum benefits from steroid has been observed and symptoms remains unsatisfactory. Exclusion criteria includes history of foot and ankle trauma, peripheral vascular disease, presence of infection or wound, diabetic foot complications, osteoarthritis of the foot, and steroid injection within one month of surgery. Patients with systemic inflammatory arthritis, neuropathy, and pregnancy would also be excluded. EPFR surgeries were performed by a single senior consultant via two portal technique. This study was approved by authors’ affiliated institution’s ethical committee and informed consent was taken from patients.

**Fig. 1:**
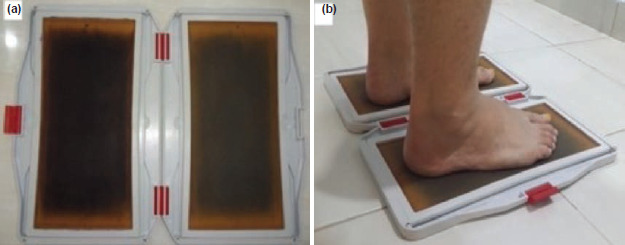
(a). Harris-Beath footprint mat. (b) Acquiring footprint.

**Fig. 2:**
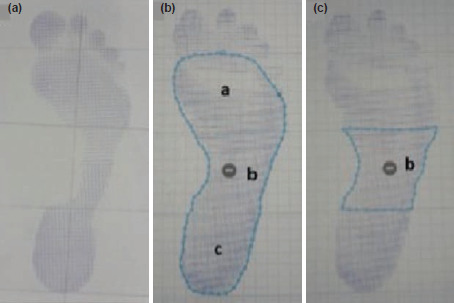
(a) Raw footprint. Foot axis drawn and foot length divided into three equal portions. (b) Software calculated total area. (c) Software calculated midfoot area. Arch Index is taken as b/ (a+b+c).

Patients’ footprints were obtained from Harris-Beath footprint mat, which consists of two rectangular plates with a rubber sheet. Under surface of the rubber sheet was structured with square grids, to which water-soluble ink was applied. A piece of white paper was placed under each rubber sheet to register the footprint. This was taken for both feet, in static, bipedal stance position and with full weight-bearing (Fig. 1). On the footprint, a line termed “foot axis” was drawn from second metatarsal to the centre of the heel, and two perpendicular lines drawn to this axis, one at the most posterior aspect of the heel and one at the most anterior aspect of the footprint excluding the toes. The area between these two lines was then divided into three equal parts, thus forefoot, midfoot and hind foot. Arch index was defined by the area of midfoot to the ratio of total area (excluding the toes). Arch index of ≤0.21 was indicative of cavus foot (high arch), ≥0.26 was indicative of a planus foot (flat arch)^17^ (Fig. 2).

The footprints and arch index were taken and calculated pre-operatively and at 12 months post-surgery to assess changes in the medial longitudinal arch of the feet. Footprints were scanned into a software [The SketchAndCalc^®^™], corrected to actual size ratio, and area calculated. Each footprint was processed three times and the average reading was taken.

**Fig. 3:**
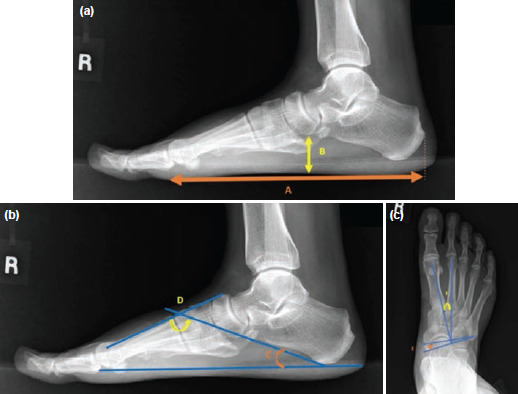
(a) [A] Measurement of truncated foot length and [B] navicular height. Normalised navicular height truncated, NNHt, is B/A. (b) Measurement of calcaneal inclination angle, [C] CIA and [D] Calcaneal 1st metatarsal angle, C1MA. (c) Measurement of Talonavicular coverage angle, [E] TNCA and [F] Talus 2nd metatarsal angle,T2MA.

Standardised anteroposterior (AP) and lateral weight bearing radiographs were taken on relaxed bipedal stance position for each patient, pre-operatively and 12 months after surgery. From the lateral view, normalised navicular height (NNHt), calcaneal inclination angle (CIA) and calcaneal-first metatarsal angle (C1MA) were measured (Fig. 3a and b). From the AP view, talus-second metatarsal angle (T2MA) and talo-navicular coverage angle (TNCA) were measured (Fig. 3c). The reliability of these measurements has been reported to be moderate to excellent^18^. NNHt is a reliable index to quantify the medial longitudinal arch^19^. True navicular height is the distance measured from the navicular tuberosity to the supporting surface. Normalised navicular height truncated is the ratio of true navicular height corrected to the truncated length of the foot – first metatarsal head to the most posterior aspect of calcaneum. Normal value is 0.17-0.19, value <0.17 indicates flat arch foot. CIA is the tangent measured from the supporting surface to the inferior border of calcaneum. Normal angle is 18° - 30°, value <18° indicates flat arch foot. C1MA is formed by the inferior border of the calcaneum, and a line drawn along the dorsum midshaft of first metatarsal. Normal angle is 130° - 136°, value >136° indicates flat arch foot. T2MA is formed by bisection of second metatarsal and a line perpendicular to a line drawn connecting anteromedial and anterolateral extremes of talar head. Normal angle is <16°, value >16° indicates flat arch foot. TNCA is formed by a line connecting the anteromedial and anterolateral extremes of the talar head and bisection of the proximal articular surface of the navicular. Normal angle is <7°, value >7° indicates flat arch foot. These five measurements together with AI were recognised as a screening protocol in classifying foot posture in research studies^20^.

**Fig 4:**
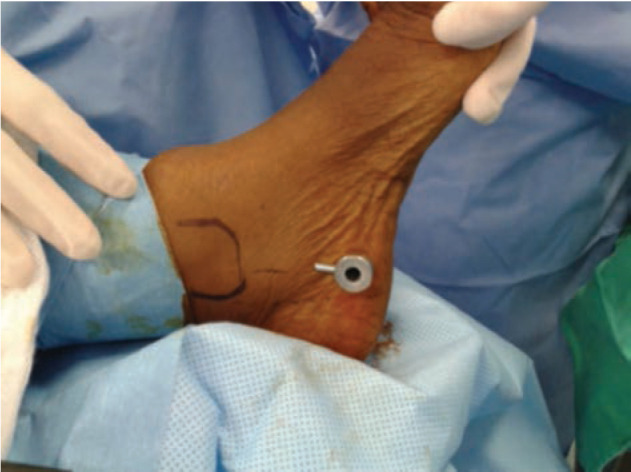
Anatomical landmark for medial portal. Along the midline of medial malleolus and approximately 15mm from plantar surface.

**Fig 5:**
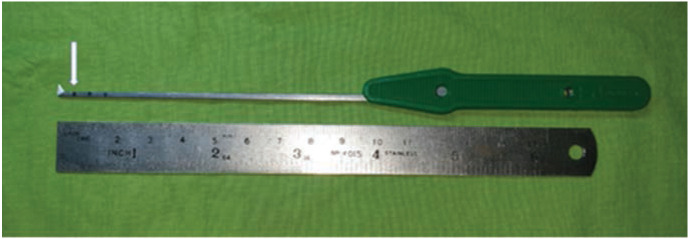
5mm, 10mm and 15mm (arrow) marked from the tip of hook blade.

**Fig. 6:**
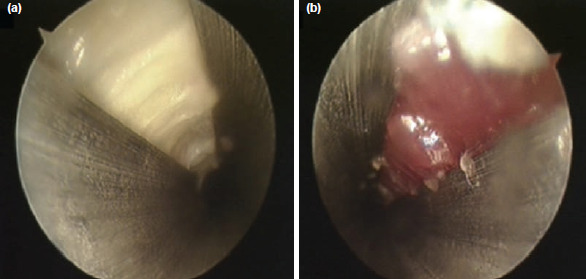
Endoscopic view (a) Intact plantar fascia prior to release, (b) after medial released. Intrinsic muscles left intact.

All the procedures were performed by a single surgeon using endoscopic two-portal system [Dyonis Ectra II Ligament release system, Smith & Nephew, MA, USA.]. The surgical set consisted of a fascial elevator, an obturator, cannula, rasp, and a disposable hook blade. The patient was placed in supine position, with a sandbag support under the ipsilateral gluteus. The limb was prepared and positioned with the foot and toes pointing upwards. A 5mm vertical incision made on the point where midline of medial malleolus and approximately 15mm superior from the heel intersect (Fig. 4). Gentle blunt dissection performed and the fascia elevator advanced inferior to the plantar fascia medial to laterally. Once its tip reached the lateral border of the foot, another 5mm vertical incision made for the lateral portal. Upon removal of the elevator, the elevator was used to strum on the fascia to ensure the elevator was inferior to the fascia. Obturator together with the cannula was then inserted along the path created by the elevator. The obturator was then removed, and the cannula left in place. The cannula allowed direct visualisation of the plantar fascia. The rasp was used to remove excessive fat if necessary. The endoscope was then introduced into the medial portal to identify the plantar fascia band. The hook blade was marked at 5mm, 10mm and 15mm from the tip (Fig. 5). The whole width of plantar fascia was visualised, the hook blade was introduced via lateral portal and used as a guide to mark 50% or centre of the plantar fascia. The fascial was release from medial to lateral, with first metatarsophalangeal joint in dorsiflexion, until the medial tightness was sufficiently released by palpation on medial sole. Attention was given to the marking not to exceed 50% of the width of plantar fascia. The release was confirmed by seeing the underlying muscle belly of the intrinsics (Fig. 6). By releasing not more than half or 50% of the total width, the lateral band of plantar fascia was left intact. The instrumentation was then removed, and wound closed with 3-0 nylon suture. A small compressive dressing applied.

Adequate analgesics were given, and patients were advised for non-weight bearing for two weeks, followed by activity as tolerated. The period to full weight-bearing varies among patients. The wounds were inspected after three days, and the sutures were removed two weeks post-operatively.

Standard statistical analysis was performed using IBM SPSS statistics version 26.0 [IBM, Armonk, NY]. For comparison of variances in two groups (pre-op and post-op arch index and radiological measurements), paired-sample T-test was used. A p-value of less than 0.05 (95% confidence interval) was considered significant.

## Results

We recruited 16 patients (11 females, 5 males) with 18 feet for the EPFR. Ten were on the right feet, while four were on the left. Two patients (both females) had both feet treated at an interval at least three months apart from the first foot surgery. Age of the patients ranged from 29 to 71 years (mean±SD, 47.72±10.69). All of them were followed-up from 14 to 31 months after surgery (23.44±5.76). The mean body mass index (BMI) (±SD) was 29.35 (±6.09) kg/m^2^, with range from 19.56 kg/m^2^ to 43.69 kg/m^2^. There were 6 patients with BMI ≤25 kg/m^2^, 7 patients were overweight (BMI 25-30 kg/m^2^), and 3 patients were obese (BMI ≥30 kg/m^2^). In relation to the occupation of the patients, there were six teachers, four healthcare workers, three administrative workers and one each was engineer, postman and housewife. The duration of symptoms prior to surgery ranged from 6 months to 10 years, with a mean of 32.67 (±30.12) months. All patients underwent physiotherapy for stretching exercises, night splint and NSAIDs starting at least six months prior to surgery. In addition to that, 6 feet had local steroid injections, one foot had PRP injection, and one had acupuncture therapy (Table I).

**Table I:** Demographic and perioperative data

Parameters (n =16)	
Gender	
Male	5 (31.25%)
Female	11 (68.75%)
Age (years)	
Mean (±SD)	47.72 (±10.69)
Median (range)	46.50 (29 - 71)
Side	
Left	4 (25.0%)
Right	10 (62.5%)
Both	2 (12.5%)
BMI (kg/m2)	
≤ 25	6 (37.5%)
25 - 30	7 (43.75%)
≥30	3 (18.75%)
Mean (±SD)	29.35 (±6.09)
Median (range)	29.83 (19.56 - 43.69)
Duration of symptoms (months) (n=18)	
Mean (±SD)	32.67 (±30.12)
Median (range)	24.0 (6 - 120)
Treatment prior to surgery* (n=18)	
Steroid injection	6 (37.5%)
Platelet-rich plasma injection	1 (6.25%)
Acupuncture	1 (6.25%)
Duration of follow-up (months) (n=18)	
Mean (±SD)	23.44 (±5.76)
Median (range)	25.0 (14 - 31)
Occupation	
Teacher	6 (37.5%)
Healthcare worker	4 (25.0%)
Administrative officer	3 (18.75%)
Others	3 (18.75%)

* in addition to physiotherapy, night splint and non-steroidal anti-inflammatory drugs.

Arch Index and radiographs data were normally distributed. The overall mean Arch Index (AI) before surgery was 0.27 (±0.05), and it was 0.29 (±0.05) on last clinical review. The increase in AI before and after surgery was statistically significant (p<0.05) (Table II). According to AI measurement, there were 7 patients with flat arch feet and 11 normal arch feet before surgery, but on the last follow-up 2 patients from normal arch feet progressed to flat arch feet. We grouped the patients into normal arch and flat arch pre-surgery and analyse the changes of AI after surgery in these two groups of patients. We found that the increased in AI in normal arch patients was statistically significant (p<0.05), in comparison to flat arch patients was not statistically significant (p=0.051) (Table III).

**Table II:** Arch index and radiograph measurements pre- and post-surgery

	Pre-surgery (mean±SD) n=18	Post-surgery (mean±SD) n=18	*P* value
AI	0.27 (± 0.05)	0.29 (±0.05)	***0.004***
NNHt	0.13 (±0.03)	0.12 (±0.03)	0.539
CIA(°)	20.37 (±5.52)	18.61 (±5.83)	***0.031***
C1MA(°)	135.61 (±7.54)	138.56 (±7.48)	***0.031***
TNCA(°)	10.78 (±5.72)	13.44 (±5.93)	0.064
T2MA(°)	10.67 (±5.75)	13.06 (±6.82)	0.098

*Abbreviations* - AI: arch index, NNHt: normalised navicular height truncated, CIA: calcaneal inclination angle, C1MA: calcaneal-first metatarsal angle, TNCA: talo-navicular coverage angle, T2MA: talus-second metatarsal angle

**Table III:** Comparison of Arch Index changes in normal arch group and flat arch group pre- and post-surgery

	Pre-surgery Arch Index (mean±SD) n=7	Post-surgery Post-surgery (mean±SD) n=11	Changes in Arch Index (mean±SD)	P value
Normal arch group	0.24 (±0.04)	0.26 (±0.04)	0.022 (±0.029)	***0.034***
Flat arch group	0.32 (±0.02)	0.34 (±0.03)	0.018 (±0.019)	0.051

The mean Normalised Navicular Height truncated (NNHt) before surgery was 0.13 (±0.03), at 12 months after surgery it was 0.12 (±0.03). The mean Calcaneal Inclination Angle (CIA) reduced from 20.37(±5.52)° before surgery to 18.61(±5.83)° at 12 months after surgery. The mean Calcano-1st Metatarsal Angle (C1MA) increased from 135.61(±7.54)° before surgery to 138.56 (±7.48)° at 12 months after surgery. As for the mean Talo-navicular Angle (TNCA) it was 10.78 (±5.72)° before surgery and at 12 months after surgery it was 13.44 (±5.93)°. The mean Talo-2nd Metatarsal Angle (T2MA) showed similar pattern of change from 10.67 (±5.75)° before surgery to 13.06 (±6.82)° after surgery. Among these footprint and radiographic parameters, increment in AI, C1MA and reduction in CIA were statistically significant (p<0.05) (Table II). There was no significant correlation between age, BMI, and the changes of foot posture indices. Therefore, the null hypothesis was rejected.

One patient experienced medial calcaneal nerve entrapment symptom at nine months after surgery. After surgical release of the nerve, the symptom resolved and remained asymptomatic up to 29 months of follow-up. One patient had fifth metatarsal bursitis three months after surgery, and another one had fourth and fifth metatarsal pain at six months after surgery. Both responded well with non-operative treatment which consisted of rest, stretching exercises, orthotics, and non-steroidal anti-inflammatory drugs. They were observed up to 26- and 29-months post EPFR, respectively.

## Discussion

Several causes have been hypothesised as the aetiology of plantar fasciitis. However, in approximately 85% of the cases, the underlying cause remains unknown^21^. Among the common risk factors reported were age >40 years old, obesity, occupations involving prolonged weight bearing, pes planus or pes cavus, and tightness or weakness of the calf muscles^22-24^.

In our series, patients were predominantly female, with female to male ratio 2.2:1. This correspond to findings in most studies^25^. BMI has been shown to be a risk factor with plantar-fasciitis, and 62.5% of our patients were either overweight or obese. Eleven patients were involved in occupations which required prolonged weight-bearing, i.e., six were teachers, four were healthcare workers and one was a postman.

Defining foot posture has been a complicated effort in the history of podiatric and Orthopaedics. The gold standard of medial longitudinal arch (MLA) assessment was radiological measures^26^. Footprint analysis was also used as indirect evaluation of MLA. We incorporated both footprints and radiological measurements in this study for comprehensive assessment of MLA. After section of medial plantar fascia, in vivo healing of the lengthened fascia takes place between six to eight weeks. Therefore, repeated measurements were taken at 12 months to allow realistic loading of the foot as patients return to their normal activity level. Our study showed after EPFR procedure, there is an increase in AI and C1MA, reduction in CIA (p<0.05), indicating a decrease in height medial longitudinal arch. Two feet progressed from normal arch to flat arch after surgery without any symptoms. The two patients did not notice their foot posture changes nor increase in foot size. Interestingly, our data showed that patients with pre-surgery normal arch feet experienced more significant changes in AI comparing to flat arch patients. Despite being statistically significant in changes of MLA indices, the changes was in single digit or decimals that might not have been manifested clinically in daily activities. Efficacy of EPFR has been affirmed since its introduction in the 1990’s. However, there remained some controversy on the biomechanical integrity of the foot and long-term outcome following surgery. By releasing the plantar fascia, surgeons inevitably alter the biomechanics of the foot. Plantar fascia is a thickened fibrous aponeurosis that originates from medial calcaneal tubercle, attached distally to five digits at proximal phalanges each by separate bundles of tissue. It acts as a tie-rod across the calcaneum to the phalanges and prevents foot collapse by its anatomical orientation and tensile strength. It simulates a ‘windlass’ that attached to the calcaneum and the metatarsophalangeal joints. Passive dorsiflexion of the metatarsal during propulsive phase of the gait stretches the plantar fascia and causing its shortening. This results in higher foot arch and the mid-tarsal bones are more stable for more efficient push-off by the toes. This action is known as the Hick’s Windlass Mechanism^27^. The stability of MLA is contributed by plantar fascia, long and short plantar ligaments, plantar calcaneonavicular ligament, interlocking of tarsal bones and to a lesser extent by joint capsules, intrinsic and extrinsic muscles. Sectioning of plantar fascia in early 1990s reported good to excellent pain relief but also drew critical evaluation to its possible complications especially in complete release cases, where weakness of MLA (failure in absorbing compressive force) causing altered forefoot and second metatarsal loading, increasing force acting on lateral and medial columns of the foot^28^.

In 1992, Daly *et al*^9^ reported on a series of open plantar fasciotomy and observed flattening of longitudinal arch, changes in lateral medial forces, and tendency of patients to avoid heel loading. Arangio *et al*^8^ noted that there was 17% vertical displacement and 15% horizontal elongation of the foot after total release of plantar fascia. Barrett *et al*^29^ reported 12 complications in their series of 65 cases, 9 were related with to lateral column pain with 6 patients having pain at the cuboid, while 2 had painful os perineum. He first introduced the word “lateral” and “medial column destabilisation phenomena”^10^. Medial column destabilising phenomena or syndromes comprises of medial column pathology such as medial plantar nerve neuropathy secondary to increased pressure in the medial plantar tunnel. On the other hand, lateral column destabilisation phenomena consist of calcaneo-cuboidal pain, lateral midtarsal pain, 4th and 5th metatarsal pain or bursitis, sinus tarsi syndrome, os peroneum pain and peroneal tenosynovitis. These changes reflect the increased loading on the lateral column of the foot. This phenomenon usually presents around third week post-surgery and seldom present later than eight weeks.

Pertaining to the biomechanical destabilising problem following plantar fascia release, the ideal percentage for release of plantar fascia remains controversial. In reporting his complications in 1993, Barrett *et al*^29^ recommended that in order to reduce lateral column problems, transaction of lateral band of plantar fascia should be avoided and replaced with two-thirds release. Stone and McClure, and Thordarson *et al* recommended a partial release from 33-66%^30,31^. Brugh *et al*^12^ in 2002 showed that there was a significant increase in lateral column pain and instability when more than 50% of the plantar fascia was released. They suggested that 50% would be a balance between eradication of heel pain and risk of lateral column pain. Based on these literatures and finite element model study by Cheung *et al*^11^, we released less than half (50%) of the plantar fascia on the medial aspect to balance between satisfactory symptoms relief and complication.

We encounter three complications in our series. One patient required second surgery nine months after EPFR to explore and release of medial calcaneal branch of posterior tibial nerve, after developing medial heel pain. We believed this was due to increased pressure in the medial ankle tunnels following plantar fascia release, similar to Barrett^4^, reported this as one of the medial column syndromes. This patient, however, had no significant foot arch changes. Two patients reported lateral column destabilisation symptoms and were managed conservatively. Symptoms were relieved fully after four to six weeks. These two patients were not associated with significant foot arch changes. Our observation suggested that symptomatic complications following EPFR may not be associated with physical changes in the foot arch. The main limitation of this study is the small sample size. A larger study with a control group (conservative management) or open plantar fasciotomy would provide comparison of foot arch changes. The biomechanics of foot varies dynamically during different gait phases, while radiographic parameters and footprint measurements only reflect the foot in static position. Gait study with pedobarograph analysis may be able to provide additional information on the loading patterns of the whole gait cycle post EPFR.

## Conclusion

Our study showed that EPFR is associated with asymptomatic reduction in MLA. We recommend EPFR as an option of treatment for chronic recalcitrant plantar fasciitis, with not more than 50% of plantar fascia release. Similar to open plantar fascia surgery, destabilising symptoms can present early as three months and late around six to nine months post-surgery. These symptoms were self-limiting. We suggest patients’ follow-up should be continued at least for 12 months and longer for those with symptoms.
